# Intermittent Iron Folate Supplementation: Impact on Hematinic Status and Growth of School Girls

**DOI:** 10.5402/2012/482153

**Published:** 2012-07-26

**Authors:** Aditi Sen, Shubhada Kanani

**Affiliations:** Department of Foods and Nutrition, The Maharaja Sayajirao University of Baroda, 14 Anupam Society, Behind Pizza Inn, Jetalpur Road, Vadodara 390007, India

## Abstract

In view of high iron needs for adolescent growth, this paper studied the impact of daily vs. intermittent (once and twice weekly) iron folic acid (IFA) supplementation on hemoglobin levels and pubertal growth among primary school girls in early adolescence (9–13 years) of Vadodara, India. *Methods*. Hemoglobin (Hb), height and weight of the girls were assessed using standard methods. In three experimental schools (ES) IFA tablets in a dose of 100 mg Fe + 0.5 mg folic acid was given either daily, once weekly or twice weekly for one year. The fourth school (control: CS) did not receive any intervention. *Results*. Hb levels significantly improved (*P* < 0.01) in all ES compared to CS. Body Mass Index (BMI) increment in ES vs CS was significant (*P* < 0.05) in twice weekly IFA and daily IFA. Within ES groups, mean Hb and BMI increments were comparable between twice weekly IFA and daily IFA. Anemic ES girls showed higher Hb and BMI increments vs. non-anemic girls. Better the Hb response, greater was the benefit on BMI. Conclusion: Twice-weekly IFA supplementation was comparable to daily IFA as regards impact on Hb and growth; at less cost and greater feasibility. Once-weekly dose was inadequate to significantly improve growth.

## 1. Introduction

Iron-deficiency anemia (IDA) leads to deleterious effects not only on hematinic status, but it may also adversely impact physical-mental development and school performance. The adverse impact on growth is of particular concern. The period of preadolescence and early adolescence (9–13 years) is a vulnerable period for anemia due to the higher nutrient demands for rapid growth spurt. Marginal iron intakes may lead to aggravation of existing anemia in young adolescents due to a higher demand of iron during the growth period. Conversely, low iron status may limit their growth spurt [1]. IDA may suppress appetite in anemics [2], leading to poor food intake, again adversely influencing adolescent growth. Research in urban and rural Indian communities has shown a high prevalence of anemia (about 90%) among adolescent girls (hemoglobin (Hb) <12 g/dL) [3, 4], and a majority are growth-retarded showing weight and BMI deficits in early adolescence [3, 5]. Small body size has long-term adverse consequences on pregnancy outcomes in terms of low birth weight, risk of obstructive complications and maternal mortality. Above 50% of the women are already anemic before they become pregnant [4, 6]. Early marriage and early pregnancy, common in India, increase the vulnerability of girls to obstetric complications and mortality risk. 

Daily iron folic acid supplementation has shown positive impact on hematinic status as well as growth [2, 7], but little is known of the growth impact of *intermittent *iron supplementation on growth. As suggested by the mucosal block theory, weekly or intermittent iron supplementation may show overall similar iron absorption over the week as compared to daily supplements [8]. Once-weekly supplements have led to significant benefits on Hb levels among adolescent girls, which was comparable to benefits experienced due to daily supplements [8]. Similar impact was seen when once weekly iron folate supplements were given in school settings [9]. The advantage of school settings is that supervised distribution of the IFA dose is possible. Weekly supplements have also reported fewer side effects, increased compliance, and reduced cost. 

In the state of Gujarat in western region of India, the Government of Gujarat has been implementing the *Adolescent Girls' Anemia Reduction Program* for over a decade now in all the secondary and higher secondary schools, wherein each girl is given once weekly iron folic acid (IFA) tablet under supervision of class teacher or class monitor, and significant impact was reported on hemoglobin levels in the impact analysis of the pilot trial [10]. However, inadequate data is available on the impact of weekly dosing on growth, especially in younger adolescents who are the pubertal phase of rapid growth. Our exploratory research in urban Vadodara showed impact of weekly supplementation on Hb levels but no significant impact on the growth variable [3]. Could twice-weekly supplementation over one school year show a better impact on growth and anemia reduction among school girls? Just as older girls in secondary schools are receiving IFA under the Government program, will intermittent supplements particularly benefit younger girls in early adolescence in primary schools by reducing anemia and improving growth? This paper reports a study conducted in urban primary schools to answer these research questions. The central objective was to investigate the relative impact of daily, once-weekly, and twice-weekly IFA supplementation on Hb levels and BMI among young school going adolescent girls.

## 2. Material and Methods

This experimental-control semilongitudinal study; an efficacy trial to assess impact of iron folic acid supplements on hematinic status and growth, was conducted in Vadodara municipal primary schools catering to girls from low-income group families (LIG). From a list of all schools under the Corporation, schools matching the following criteria were selected as the universe for sampling: schools having only girl students; having a primary section catering to 9–13 year old girls in grades V and VI, and similar school timings (morning shift). From a sampling frame of 17 government urban primary schools thus identified, four schools were randomly selected, and all the girls studying in grades V and VI were enrolled in the study. Prior permission from the Primary School Board, Vadodara, was taken, and the schools were explained the purpose of the study. Informed written consent was taken from the girls and their parents. The students were free to opt out of the study anytime they wanted. Permission was obtained from all students of classes V and VI who comprised the sample.

The four schools were randomly assigned to three experimental schools (ES) and were given IFA tablets (100 mg elemental iron + 0.5 mg folic acid) either once weekly (E1: IFA-1Wkly) or twice weekly (E2: IFA-2Wkly) or daily (ED: IFA-Daily) for one year. The fourth was the control school (CS: No-IFA) which did not receive any intervention. The class monitor/class teacher distributed IFA and maintained compliance registers. The investigators with assistance from the class teachers/monitors ensured regular supervised distribution and recording of compliance in all the ES schools. The tablets were distributed immediately after the tiffin break in all the schools so that they are not consumed on empty stomach. 

*Sample size calculation* was done to estimate how many schools would be required for the study assuming that there are 100 girls in standards V and VI in each school (based on our exploratory visits). Using standard formulae for estimating sample size [11] for a study such as this, where each treatment group is compared pre and post intervention, and is also compared with the control; the desired sample size came to 46 subjects per group. Assuming the proportion of girls with anemia as 60% at baseline, a difference of 20% after the intervention and significance level of 0.05; also allowing for dropouts, it was decided that each study group (representing one school) would have about 60 subjects. Since one school on an average had approximately 60–100 girls available, it was clear that one school would give enough sample size for one study group. As there were four groups (three experimental and one control), it was decided that four schools would be randomly sampled for this study.

Blood sample from finger prick was collected from all girls willing and available in V and VI standards, before and after the intervention which was assessed for hemoglobin status using cyanmethemoglobin method [12]. Standard methods were used for height, and weight [13]. Data on Hb, height and weight (BMI) are reported here on all girls available in V and VI standards both before and after the supplementation period (*N* = 254). Girls who had attained their menarche (*N* = 18) prior to, or, during the study were excluded from the analysis, though they did receive IFA supplements. Body mass index was calculated using the standard formula (weight (Kg)/height (m^2^)). Percentage of anemic girls was calculated using WHO cutoff of 12 g/dL Hb. Undernutrition was defined as BMI < 5th percentile of Must et al. standards [14]. The undernutrition profile thus obtained was comparable to that obtained from WHO (2007) standards; further, the focus was on comparing relative impact between ES and CS; within the three ES groups. 

### 2.1. Statistical Analysis

 All the data were coded, entered, and analyzed in Epi Info, version 6.04-d [15]. All analysis was done on the final data set comprising only those girls for whom data was available at both the preintervention and postintervention phases. The changes in each indicator of impact (pre to post) in each group (ES and CS) were measured and compared for increments in hemoglobin levels and growth (body mass index). Initially anemics versus initially nonanemics were also compared: experimental schools (ES) versus control school (CS). As appropriate, either *F*-test or Student's *t*-test was used to compare various intervention groups for statistical significance of impact. 

## 3. Results

### 3.1. Profile of Study Participants

Majority of the girls were in the age group of 9 to 13 years. Nearly half (46.2%) of the mothers were illiterate, and most of the fathers (81.0%) were educated only till primary level. Fathers of the girls were employed in low-income generating services. Almost half (43.1%) of the mothers were employed as maids. The key socioeconomic indicators, as well as baseline Hb and BMI values, were statistically similar in all the four schools. 

### 3.2. Impact on Hemoglobin Levels after the Intervention Period of One Year

**Figure 1:**
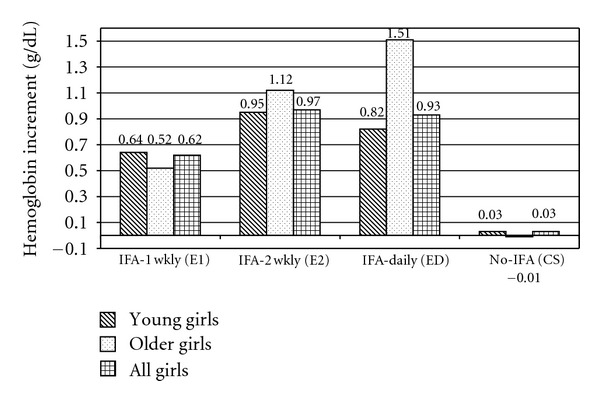
Mean increment in hemoglobin levels among younger and older girls after the intervention. In all girls: *P* < 0.001 (E1 versus CS, E2 versus CS, and ED versus CS), *P* < 0.05 (E2 versus E1),^NS^non significant (ED versus E1and E2 versus ED), IFA-1Wkly: once a week IFA tablet, IFA-2Wkly: twice a week IFA tablet, IFA-Daily: daily IFA tablet, and No-IFA: no IFA tablet.

The increment in Hb levels in all the three intervention groups was significantly higher compared to the control group. The comparative mean increment values in Hb levels among the various intervention groups (Table 1) indicate that the increment was the highest (0.97 g/dL) in the IFA twice-weekly group (IFA-2Wkly) followed by IFA-daily group (0.93 g/dL), while IFA once-weekly group (IFA-1Wkly) showed the lowest increment (0.62 g/dL). Control group showed negligible change in mean Hb levels. Within younger and older age group, the treated groups had significantly higher Hb levels than the No-IFA group (Figure 1). On comparing younger with older girls, it was seen that Hb increments in IFA-2Wkly and IFA-Daily were relatively higher in older girls. 

The mean Hb increments among *initially anemic* girls in all the supplemented groups were higher than those among *initially nonanemic* girls (Table 2). Among initially anemic girls, IFA-daily group showed highest increments (1.9 g/dL) followed closely by IFA-2Wkly (1.6 g/dL) and IFA-1Wkly (1 g/dL). In No-IFA controls, initially anemic girls showed small increment in Hb levels; while the nonanemics showed a small deterioration in Hb levels.

### 3.3. Did the Level of Compliance Influence the Impact of Iron Supplementation? 

**Figure 2:**
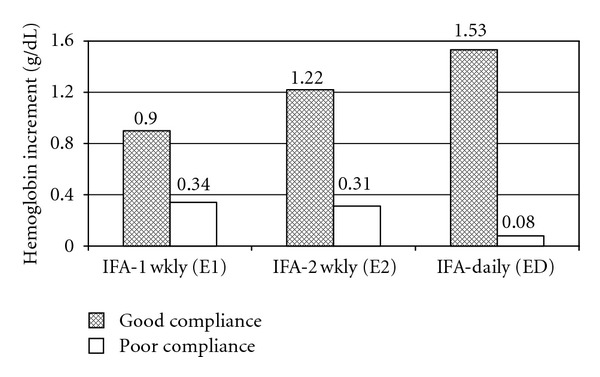
Mean hemoglobin increment in girls with good^1^ compliance and poor^2^ compliance of iron folic acid tablets. ^1^compliance of ≥70% of IFA tablets, ^2^compliance of <70% of IFA tablets, IFA-1Wkly: once a week IFA tablet, IFA-2Wkly: twice a week IFA tablet, and IFA-Daily: daily IFA tablet.

Overall, the mean compliance of IFA tablets was 72%. The girls were further categorized according to their compliance of iron folic acid tablets. Good compliance (>70% dose consumed) was seen in above 50% subjects in all 3 groups. The mean change in Hb levels was significantly better in girls with good compliance than those with poor compliance in all the intervention groups but the difference in Hb increment (0.8 to 1 g/dL Hb) was higher in the IFA-2Wkly and IFA-Daily groups compared to the less compliant IFA-1Wkly group (0.6 g/dL) (Figure 2). However, there was no significant difference among various intervention groups, and the increments were comparable. 

### 3.4. Impact on Growth in Terms of BMI after the Intervention Period of One Year

The mean change in BMI was measured after the intervention period (Table 3). There was a significant increment in BMI in all the intervention groups as well as the control group compared to baseline; however, the girls in the three supplemented groups had better BMI gains compared to the gains in the nonsupplemented group. On comparing BMI increment of various regimens with control (No-IFA group), IFA-1Wkly group showed no significant difference, whereas BMI gains were significantly higher in the IFA-2Wkly and Daily-IFA groups versus control. Among the intervention groups, the mean increment in BMI was higher in the IFA-2Wkly and IFA-Daily groups compared to once-weekly group. 

On comparing the initially anemic girls with initially nonanemic girls (Table 4) it was observed that initially anemic girls had higher BMI gains compared to their nonanemic counterparts, and these differences were statistically significant in IFA-2Wkly and IFA-Daily groups.

### 3.5. Did the Extent of Hemoglobin Gain Influence the Gain in BMI?

**Figure 3:**
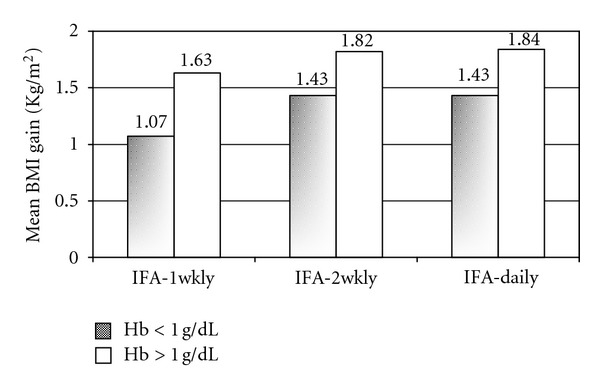
BMI gain according to change in hemoglobin levels among the Three intervened groups after the intervention. BMI: body mass index, IFA-1Wkly: once a week IFA tablet, IFA-2Wkly: twice a week IFA tablet, and IFA-Daily: daily IFA tablet.

In girls whose Hb gain was >1 g/dL, their BMI gains were higher compared to those whose Hb gain was <1 g/dL, in all treatment groups (Figure 3). Even in the girls showing <1 g/dL Hb increment, BMI gains tended to be higher in IFA-2Wkly and IFA-Daily groups, as compared to IFA-1Wkly. In No-IFA group, none of the girls gained more than 1 g/dL of Hb; therefore, this group has not been included in the figure.

## 4. Discussion 

In the present study, as compared to controls, all three dose regimens of iron folic acid supplementation (daily, once and twice weekly) significantly improved Hb levels, but growth (BMI gain) was significant only in daily- and twice-weekly IFA supplementation. Hb increments were higher in anemic girls compared to the nonanemic girls. There is scanty literature available on hematinic benefits of intermittent iron folate supplementation on young adolescent girls especially twice weekly IFA. Reported impact of daily- and/or once-weekly supplements is discussed here. 

After two months of supplementation with ferrous sulfate (200 mg) among Karachi school children (5–10 yrs) having Iron-deficiency anemia, there was a significant rise in the concentrations of Hb and hematocrit (Hct) in both daily- and once-weekly supplementation school groups [16]. A significant improvement in the Hb levels was reported in groups of adolescent girls who were supplemented different dose levels of iron folate supplements [17]. A study reported by Indian Institute of Health and Family Welfare (2001-2002) [18] examined the efficacy of once-weekly IFA supplementation among rural school going adolescent girls in Andhra Pradesh and reported beneficial effects on Hb levels in the once-weekly supplementation group. Data of the present study and reports from literature indicate that iron folate given once or twice (about 100 mg elemental iron dose) suffices to enhance the hematinic status of young adolescents—not only hemoglobin but storage iron as well.

With regard to growth,in the present study, girls showed significant increments in BMI only when treated with iron folic acid tablets either twice a week or daily. Impact was more marked among initially anemic girls. Once-weekly IFA supplementation did not significantly improve the BMI even among the anemic girls. In our earlier study, adolescent girls (10–18 yrs) in urban slums of Vadodara, receiving daily-IFA supplements for three months significantly improved the Hb levels and weight gain when compared to the controls [2]. Growth impact among anemic primary school children (6–11 yrs) has been reported in Kenya receiving sustained release ferrous sulfate (150 mg) tablets daily for 14 weeks [7].

Research elsewhere appears to suggest that once-weekly supplementation does not appear to enhance growth, though this is likely to vary according to dose levels and duration of supplementation. In Indonesia, supplementation with once weekly iron folic acid (60 mg Fe + 0.25 mg folic acid) among anemic adolescent girls (13–16 yrs) resulted in significant improvements in the Hb, but not in weight and height gain after five weeks [19]. This period is, however, too short to observe changes in growth. An urban project in Vadodara found that once-weekly IFA supplementation even over a period of one school year did not significantly improve growth, which corroborates the present study findings [3]. Our study observed significant benefits on growth after twice-weekly iron folate supplementation. The favorable impact on weight gain appears to be related to Hb gains. More frequent dosing with iron folate leading to a higher Hb levels appears to be necessary for achieving higher weight gains as suggested in a study on anemic preschool children, which reported higher rate of weight gain in the children whose hemoglobin concentration rose by at least 2 g/dL compared to those whose hemoglobin did not increase to this extent (*P*≈ 0.05) [20]. We also observed BMI gains to be higher in those who showed higher Hb increments.

The positive association of hemoglobin increments-more frequent iron dosing with better adolescent growth is explained by the marked increase in iron requirements during early adolescent growth spurt; for the expansion of total blood volume, increase in lean body mass and rapid skeletal muscle development. [21]. Total iron requirements for adolescents are computed from the increased iron requirements for the expansion of the total blood volume (0.18 mg/d in boys and 0.14 mg/d in girls on average) and the increase in the total body essential pool with the increase in the lean body mass (0.55 mg/d in boys and 0.33 mg/d in girls)—median additional requirements. The increase in the iron requirements for the red cell mass includes both the increase in total blood volume as well as the increase in the mean hemoglobin concentration from the early adolescent years through the adolescent growth spurt. The additional iron requirements for postmenarcheal girls include the additional amount of iron lost in menses beyond the growth requirements [22]; though in this study all girls were in the premenarcheal rapid growth phase.

This research also measured impact of intermittent IFA on other functions such as cognition and physical work capacity (published elsewhere) and found a similar trend; that twice-weekly IFA showed a significant and comparable impact as did the daily IFA versus the controls; while once-weekly IFA did not [23, 24]. 

Considering the benefits of twice weekly IFA supplementation not only on Hb levels but on adolescent growth, as well as physical and mental competence, it is recommended that further research be done to document the efficacy and effectiveness of such intermittent IFA supplements which reach young girls in early adolescence and to assess impact on pubertal weight and height gains and physical-mental functions—all of which will eventually help to enhance school performance and future reproductive health of these girls. 

### 4.1. Limitation of the Study

This being an efficacy trial done with time and resource constraints as part of departmental research, and also keeping in mind the academic time table of the schools included in the study, it was not possible to include a larger sample size nor increase the duration of the trial. However, we succeeded in having 50–90 subjects in the treated groups; who could be subjected to statistical analysis. Further, another limitation was that compliance to IFA varied between the schools; which was influenced by the interest and cooperation of the school teachers. Here again, we overcame this limitation by controlling for compliance during analysis and comparing girls showing good compliance in each treated school.

## Figures and Tables

**Table 1:** Change in mean hemoglobin levels in the school girls after the intervention.

Study groups	Total (9–13 yrs)				“*t*” value
	*N*	Initial	Final	Mean change ± SD
		Mean ± SD	Mean ± SD	
IFA-1Wkly (E1)	65	11.48 ± 1.06	12.09 ± 0.44	0.62 ± 0.88	E1 versus CS: 5.07^∗∗∗^ E2 versus CS: 6.89^∗∗∗^ ED versus CS: 4.82^∗∗∗^ E2 versus E1: 2.04^∗^ ED versus E1: 1.45^NS^ E2 versus ED: 0.18^NS^
IFA-2Wkly (E2)	89	11.09 ± 1.47	12.04 ± 0.41	0.97 ± 1.23	
IFA-Daily (ED)	59	11.26 ± 1.69	12.18 ± 0.55	0.93 ± 1.38	
No-IFA (CS)	41	11.54 ± 0.67	11.54 ± 0.63	0.03 ± 0.24	
(*F* value)	8.001^∗∗∗^				

^NS^nonsignificant, ^∗^ *P* < 0.05, ^∗∗∗^ *P* < 0.001, IFA-1Wkly: once a week IFA tablet, IFA-2Wkly: twice a week IFA tablet, IFA-Daily: daily IFA tablet, and No-IFA: no IFA tablet.

**Table 2:** Change in mean hemoglobin levels of initially anemic girls after the intervention.

Study groups	Initially anemic^1^ girls				Initially nonanemic^2^ girls			
	*N*	Initial	Final	Mean change	*N*	Initial	Final	Mean change
		Mean ± SD	Mean ± SD	± SD		Mean ± SD	Mean ± SD	± SD
IFA-1Wkly	46	10.93 ± 0.58	11.93±0.33	0.98 ± 0.65	19	12.79±0.76	12.48±0.41	−0.28±0.67
IFA-2Wkly	61	10.38±1.22	11.93 ± 0.42	1.55 ± 1.03	28	12.62±0.48	12.29±0.31	−0.30±0.46
IFA-Daily	34	10.07±1.18	11.95 ± 0.43	1.89 ± 1.07	25	12.87±0.55	12.55±0.35	−0.30±0.47
No-IFA	30	11.30±0.63	11.33 ± 0.59	0.50 ± 0.22	11	12.18±0.14	12.12±0.24	−0.04±0.27
(*F*-value)	30.16^∗∗∗^	0.84^NS^

^1^Hb < 12 g/dL, ^2^Hb ≥ 12 g/dL, ^NS^nonsignificant, ^∗∗∗^ *P* < 0.001, IFA-1Wkly: once a week IFA tablet, IFA-2Wkly: twice a week IFA tablet, IFA-Daily: daily IFA tablet, and No-IFA: no IFA tablet.

**Table 3:** Mean change in body mass index in the school girls after the intervention.

Study groups	Total (9–13 yrs)				“*t*” value
	*N*	Initial	Final	Mean change ± SD
		Mean ± SD	Mean ± SD	
IFA-1Wkly (E1)	73	14.63 ± 1.92	15.81 ± 1.71	1.18 ± 1.11	E1 versus CS: 0.64^NS^
IFA-2Wkly (E2)	103	14.16 ± 1.38	15.67 ± 1.66	1.55 ± 0.83	E2 versus CS: 3.11^∗∗ ^
IFA-Daily (ED)	59	14.57 ± 1.67	15.94 ± 1.85	1.37 ± 0.72	ED versus CS: 1.99^∗^
No-IFA (CS)	43	14.71 ± 1.68	15.78 ± 1.64	1.06 ± 0.87
(*F*-value)	3.86^∗∗^

^NS^non significant, ^∗^ *P* < 0.05, ^∗∗^ *P* < 0.01, IFA-1Wkly: once a week IFA tablet, IFA-2Wkly: twice a week IFA tablet, IFA-Daily: daily IFA tablet, and No-IFA: no IFA tablet.

**Table 4:** Mean change in BMI in initially anemic girls after the intervention.

Study groups	Initially anemic^1^ girls				Initially nonanemic^2^ girls			
	*N*	Initial	Final	Mean change	*N*	Initial	Final	Mean change
		Mean ± SD	Mean ± SD	± SD (A)		Mean ± SD	Mean ± SD	± SD (B)
IFA-1Wkly	46	14.34±1.53	15.71±1.53	1.41±0.90	19	15.44±2.62	16.52±2.02	1.01±1.47	1.08^NS^
IFA-2Wkly	61	14.15±1.41	15.92±1.63	1.68±0.84	28	14.06±1.76	15.37±1.56	1.29±0.81	2.17^∗^
IFA-Daily	34	14.34±1.23	15.77±1.47	1.50±0.81	25	14.89±2.14	16.11±2.27	1.10±0.65	2.03^∗^
No-IFA	30	14.39±1.33	15.49±1.47	1.18±0.79	11	15.62±2.62	16.35±1.89	0.73±1.07	1.22^NS^
(*F*-value)	3.15^∗^	1.52^NS^

^1^Hb < 12 g/dL, ^2^Hb ≥ 12 g/dL, ^NS^non significant, ^∗^ *P* < 0.05, IFA-1Wkly: once a week IFA tablet, IFA-2Wkly: twice a week IFA tablet, IFA-Daily: daily IFA tablet, and No-IFA: no IFA tablet.
